# Supplementary material for: (p)ppGpp/GTP and Malonyl-CoA Modulate Staphylococcus aureus Adaptation to FASII Antibiotics and Provide a Basis for Synergistic Bi-Therapy
Source: mBio. 2021 Feb 2;12(1):e03193-20. doi: 10.1128/mBio.03193-20 (PMC7858065; doi:10.1128/mBio.03193-20)
Supplement: FIG S1 [file mBio.03193-20-sf001.docx]

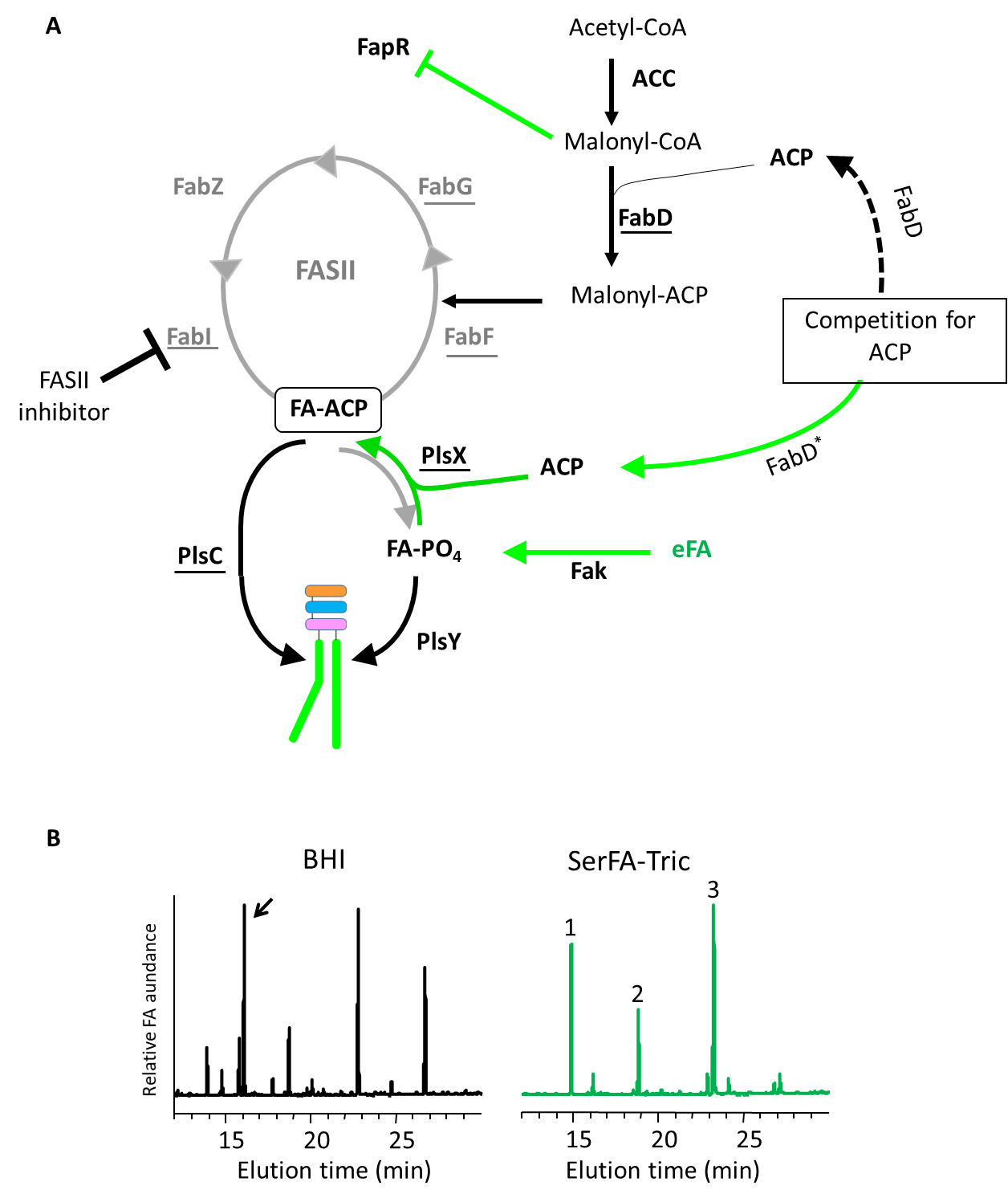


**Supplementary Fig. S1. *S. aureus* bypasses FASII inhibition by exogenous fatty acid (eFA) incorporation in membrane phospholipids. A.** Model for anti-FASII adaptation. FASII and FASII bypass are schematized as characterized; functions whose expression is controlled by FapR repressor are underlined (1-4). Malonyl-CoA reverses FapR repression (5). eFA phosphorylation by Fak (fatty acid kinase) (6) provides an intermediate that may either be incorporated in position 1 of the glycerol-3-phosphate backbone *via* PlsY, or act as a substrate for PlsX to then be incorporated in position 2 via PlsC. In the absence of serum, FabD (malonyl-CoA:ACP transacylase) mutations promote anti-FASII adaptation (2). In contrast, serum favors FASII antibiotic adaptation without FASII mutations (7). **B.** Example of fatty acid profiles of *S. aureus* Newman. Left, BHI grown cells; right, cells grown overnight in SerFA-Tric. Cultures started at *A*_600_ = 0.01 were harvested at *A*_600_ = 1. Arrow indicates *anteiso* 15 (*ai*15), the major fatty acid synthesized by *S. aureus*. eFA: 1, C14:0; 2, C16:0; 3, 18:1. Profiles are representative of three independent experiments. FA, fatty acids; FA-ACP, fatty acyl-ACP; FA-PO_4_, acyl-phosphate; grey, inhibited pathway. FabD^*^, mutated or inhibited enzyme.

1. Albanesi D, Reh G, Guerin ME, Schaeffer F, Debarbouille M, Buschiazzo A, Schujman GE, de Mendoza D, Alzari PM. 2013. Structural basis for feed-forward transcriptional regulation of membrane lipid homeostasis in *Staphylococcus aureus*. PLoS Pathog 9:e1003108.

2. Morvan C, Halpern D, Kenanian G, Hays C, Anba-Mondoloni J, Brinster S, Kennedy S, Trieu-Cuot P, Poyart C, Lamberet G, Gloux K, Gruss A. 2016. Environmental fatty acids enable emergence of infectious *Staphylococcus aureus* resistant to FASII-targeted antimicrobials. Nat Commun 7:12944.

3. Zhang YM, Rock CO. 2008. Membrane lipid homeostasis in bacteria. Nat Rev Microbiol 6:222-33.

4. Fujita Y, Matsuoka H, Hirooka K. 2007. Regulation of fatty acid metabolism in bacteria. Mol Microbiol 66:829-39.

5. Albanesi D, de Mendoza D. 2016. FapR: From Control of Membrane Lipid Homeostasis to a Biotechnological Tool. Front Mol Biosci 3:64.

6. Parsons JB, Broussard TC, Bose JL, Rosch JW, Jackson P, Subramanian C, Rock CO. 2014. Identification of a two-component fatty acid kinase responsible for host fatty acid incorporation by *Staphylococcus aureus*. Proc Natl Acad Sci U S A 111:10532-7.

7. Kenanian G, Morvan C, Weckel A, Pathania A, Anba-Mondoloni J, Halpern D, Gaillard M, Solgadi A, Dupont L, Henry C, Poyart C, Fouet A, Lamberet G, Gloux K, Gruss A. 2019. Permissive Fatty Acid Incorporation Promotes Staphylococcal Adaptation to FASII Antibiotics in Host Environments. Cell Rep 29:3974-3982 e4.
