# Supplementary material for: (p)ppGpp/GTP and Malonyl-CoA Modulate Staphylococcus aureus Adaptation to FASII Antibiotics and Provide a Basis for Synergistic Bi-Therapy
Source: mBio. 2021 Feb 2;12(1):e03193-20. doi: 10.1128/mBio.03193-20 (PMC7858065; doi:10.1128/mBio.03193-20)
Supplement: TABLE S1 [file mBio.03193-20-st001.docx]

**Table S1. Strains used in this study.**

| Strain | Phenotypes | Reference |
| --- | --- | --- |
| *S. aureus* |  |  |
| Newman | Clinical isolate | ([1](#_ENREF_1)) |
| RN4220 | *S. aureus* cloning recipient ATCC 8325-4 derivative restriction negative | ([2](#_ENREF_2)) |
| RN4220 Δ*fapR* | RN4220 derivative lacking the FapR repressor of FASII and phospholipid synthesis | ([3](#_ENREF_3)) |
| USA300 JE2 | USA300_FPR3757 strain devoid of plasmids, derived from methicillin resistant (MRSA) clinical strain | ([4](#_ENREF_4)) |
| *codY* | USA300_FPR3757 insertional mutant in *codY* (SAUSA300_1148) | ([4](#_ENREF_4)) |
| HG001 | ATCC 8325 derivative, naturally defective for *fakB1*, a component of the fatty acid kinase. | ([1](#_ENREF_1)) |
| HG001 Δ*fapR* | Strain lacking the FapR repressor of FASII and phospholipid synthesis | ([3](#_ENREF_3), [5](#_ENREF_5), [6](#_ENREF_6)) |
| HG1-R | HG001 repaired for defective *fakB1* allele | This study |
| HG1-R Δ*fapR* | HG001 Δ*fapR* repaired for defective *fakB1* allele | This study |
| HG001 ppGpp0 | Triple *rsh*, *relP*, *relQ* mutant that does not produce (p)ppGpp | ([7](#_ENREF_7)) |
| HG1-R ppGpp0  (called ppGpp0) | HG001 ppGpp0 repaired for defective *fakB1* allele | This study |
| *E. coli* |  |  |
| Top10 | F- mcrA Δ(mrr-hsdRMS-mcrBC) φ80lacZΔM15 ΔlacX74 nupG recA1 araD139 Δ(ara-leu)7697 galE15 galK16 rpsL(Str^R^) endA1 λ^-^ | Laboratory collection |
| DH5α | F^–^ *endA1* *glnV44* *thi-1* *recA1* *relA1* *gyrA96* *deoR* *nupG* *purB20* φ80d*lacZ*ΔM15 Δ(*lacZYA-argF*)U169, hsdR17(*r_K_*^–^*m_K_*^+^), λ^–^ | ([8](#_ENREF_8)) |
| IM08B | Used for direct cloning in *S. aureus* Newman | ([9](#_ENREF_9)) |
| BL21 | Used for FapR overexpression | ([3](#_ENREF_3)) |

1. Herbert S, Ziebandt AK, Ohlsen K, Schafer T, Hecker M, Albrecht D, Novick R, Gotz F. 2010. Repair of global regulators in *Staphylococcus aureus* 8325 and comparative analysis with other clinical isolates. Infect Immun 78:2877-89.

2. Kreiswirth BN, Lofdahl S, Betley MJ, O'Reilly M, Schlievert PM, Bergdoll MS, Novick RP. 1983. The toxic shock syndrome exotoxin structural gene is not detectably transmitted by a prophage. Nature 305:709-12.

3. Albanesi D, Reh G, Guerin ME, Schaeffer F, Debarbouille M, Buschiazzo A, Schujman GE, de Mendoza D, Alzari PM. 2013. Structural basis for feed-forward transcriptional regulation of membrane lipid homeostasis in *Staphylococcus aureus*. PLoS Pathog 9:e1003108.

4. Fey PD, Endres JL, Yajjala VK, Widhelm TJ, Boissy RJ, Bose JL, Bayles KW. 2013. A genetic resource for rapid and comprehensive phenotype screening of nonessential *Staphylococcus aureus* genes. MBio 4:e00537-12.

5. Kenanian G, Morvan C, Weckel A, Pathania A, Anba-Mondoloni J, Halpern D, Gaillard M, Solgadi A, Dupont L, Henry C, Poyart C, Fouet A, Lamberet G, Gloux K, Gruss A. 2019. Permissive Fatty Acid Incorporation Promotes Staphylococcal Adaptation to FASII Antibiotics in Host Environments. Cell Rep 29:3974-3982 e4.

6. Parsons JB, Broussard TC, Bose JL, Rosch JW, Jackson P, Subramanian C, Rock CO. 2014. Identification of a two-component fatty acid kinase responsible for host fatty acid incorporation by *Staphylococcus aureus*. Proc Natl Acad Sci U S A 111:10532-7.

7. Geiger T, Kastle B, Gratani FL, Goerke C, Wolz C. 2014. Two small (p)ppGpp synthases in *Staphylococcus aureus* mediate tolerance against cell envelope stress conditions. J Bacteriol 196:894-902.

8. Sambrook JRDW. 2001. Molecular cloning: a laboratory manual. 3rd ed, Cold Spring Harbor Laboratory, Cold Spring Harbor, NY.

9. Monk IR, Tree JJ, Howden BP, Stinear TP, Foster TJ. 2015. Complete Bypass of Restriction Systems for Major *Staphylococcus aureus* Lineages. MBio 6:e00308-15.
