# Supplementary material for: (p)ppGpp/GTP and Malonyl-CoA Modulate Staphylococcus aureus Adaptation to FASII Antibiotics and Provide a Basis for Synergistic Bi-Therapy
Source: mBio. 2021 Feb 2;12(1):e03193-20. doi: 10.1128/mBio.03193-20 (PMC7858065; doi:10.1128/mBio.03193-20)
Supplement: TABLE S2 [file mBio.03193-20-st002.docx]

**Table S2. Plasmids and constructions.** *^a^*

| **Plasmid** | **Description** |
| --- | --- |
| pTCV-*lac* | Promoter expression vector comprising pACYC184 and pAMβ1 as replication origins functional in respectively Gram- negative and positive bacterial hosts ([1](#_ENREF_1)). |
| pAW8 | *S. aureus* ori-pAMα1–*E. coli* ori-colE1 tetracyline-resistant shuttle vector ([2](#_ENREF_2)). |
| pG1 | Thermosensitive plasmid used to generate chromosomal gene replacement ([3](#_ENREF_3), [4](#_ENREF_4)). |
| pG1Ω*fakB1* | pG1 into which a 1,939-bp DNA encoding a functional *fakB1* was cloned by Gibson Assembly. |
| pET-21b | Cloning vector for FapR overexpression in *E. coli* ([5](#_ENREF_5)). |
| pJJ004  **(FapR-Trap)** | pTCV-*lac* derivative in which *lacZ* transcription is driven by a synthetic promoter designed from the *fabH fabF* (NWMN_0853 and NWMN_0854) operon. An 81 bp hybrid primer-pair (FapRtrapfd and FapRtraprp) was cloned into pTCV-*lac* into EcoRI and BamHI sites. A consensus FapR binding site is present in the promoter (Fig. S2). |
| pJJ005  **(P*_ilvD_*-*lacZ*)** | pTCV-*lac* derivative carrying *lacZ* fused to the promoter region of *ilvD*-*ilvB* (NWMN_1960 to NWMN_1961). A 515 bp fragment from -500 to +15 with respect to the *ilvD* start codon (genome positions 2168952-2169466) is cloned into pTCV-*lac* EcoRI-BamHI sites. The clone carries an A to G substitution at position-277. This nucleotide change is expected not to alter (p)ppGpp regulation, which occurs *via* CodY ([6](#_ENREF_6)). |
| pJJ006  **(P*_oppB_-lacZ*)** | pTCV-*lac* derivative carrying *lacZ* fused to the promoter region of the *oppB* *oppC* *oppD* *oppF* *oppA* (NWMN_0856 to NWMN_0860) operon. A 514 bp fragment from -499 to +15 with respect to the *oppB* start codon (genome positions 950296-950809) is cloned into pTCV-*lac* EcoRI-BamHI sites. |
| pJJ008  **(P*_cshA_-lacZ*)** | pTCV-*lac* derivative carrying *lacZ* fused to the *cshA* (NWMN_1985) promoter region. A 515 bp fragment from -500 to +15 with respect to the *cshA* start codon (genome positions 2207332-2207846) is cloned into pTCV-*lac* EcoRI-BamHI sites. |
| pJJ013  **(P*_fapR plsX_-lacZ*)** | pTCV-*lac* derivative carrying *lacZ* fused to the promoter region of the *fapR* *plsX* *fabD* *fabG* (NWMN_1138 to NWMN_1141) operon. A 315 bp fragment from -300 to +15 with respect to the start codon (genome positions 1247750-1248064) of the operon is cloned into pTCV-*lac* EcoRI-BamHI sites. |
| pJJ019  **(P*_plsC_-lacZ*)** | pTCV-*lac* derivative carrying *lacZ* fused to the *plsC* (NWMN_1620) promoter region. A 315 bp fragment from -300 to +15 with respect to the *plsC* start codon (genome positions 1799913-1800227) is cloned into pTCV-*lac* EcoRI-BamHI sites. |
| pJJ027  **(P*_accBC_-lacZ*)** | pTCV-*lac* derivative carrying *lacZ* fused to the promoter region of the *accB accC* (NWMN_1432, NWMN_1431) operon. Hybrid primer-pair of 103 bp (accBCfp and accBCrp), corresponding to genome positions 1604036-1603934**,** was used to clone the promoter region upstream of *accB* into pTCV-*lac* into EcoRI and BamHI sites. |
| pJJ042  (FapR-ORF) | pET21-b derivative carrying *fapR* ORF (NWMN_1138). An N-terminus hexa-histidine tag followed by a TEV cleavage site is fused to FapR. Here, Nhe1 and Sal1 sites are used for cloning. Cloning strategy is as described([5](#_ENREF_5))**.** |
| pJJ043 (P*_accBC_-lacZ*) | A pAW8-modified derivative where the *accBC*-*lacZ* fusion was amplified from pJJ027 and cloned into EcoR1 and Sma1 sites of pAW8. |

*^a^* Designations of promoter fusions are in bold (in parentheses). See Table S6 for primer pairs used for clonings.

1. Poyart C, Trieu-Cuot P. 1997. A broad-host-range mobilizable shuttle vector for the construction of transcriptional fusions to beta-galactosidase in gram-positive bacteria. FEMS Microbiol Lett 156:193-8.

2. Katayama Y, Zhang HZ, Hong D, Chambers HF. 2003. Jumping the barrier to beta-lactam resistance in Staphylococcus aureus. J Bacteriol 185:5465-72.

3. Mistou MY, Dramsi S, Brega S, Poyart C, Trieu-Cuot P. 2009. Molecular dissection of the secA2 locus of group B Streptococcus reveals that glycosylation of the Srr1 LPXTG protein is required for full virulence. J Bacteriol 191:4195-206.

4. Biswas I, Gruss A, Ehrlich SD, Maguin E. 1993. High-efficiency gene inactivation and replacement system for gram-positive bacteria. J Bacteriol 175:3628-35.

5. Albanesi D, Reh G, Guerin ME, Schaeffer F, Debarbouille M, Buschiazzo A, Schujman GE, de Mendoza D, Alzari PM. 2013. Structural basis for feed-forward transcriptional regulation of membrane lipid homeostasis in Staphylococcus aureus. PLoS Pathog 9:e1003108.

6. Kaiser JC, King AN, Grigg JC, Sheldon JR, Edgell DR, Murphy MEP, Brinsmade SR, Heinrichs DE. 2018. Repression of branched-chain amino acid synthesis in Staphylococcus aureus is mediated by isoleucine via CodY, and by a leucine-rich attenuator peptide. PLoS Genet 14:e1007159.
