# Supplementary material for: (p)ppGpp/GTP and Malonyl-CoA Modulate Staphylococcus aureus Adaptation to FASII Antibiotics and Provide a Basis for Synergistic Bi-Therapy
Source: mBio. 2021 Feb 2;12(1):e03193-20. doi: 10.1128/mBio.03193-20 (PMC7858065; doi:10.1128/mBio.03193-20)
Supplement: FIG S3 [file mBio.03193-20-sf003.docx]

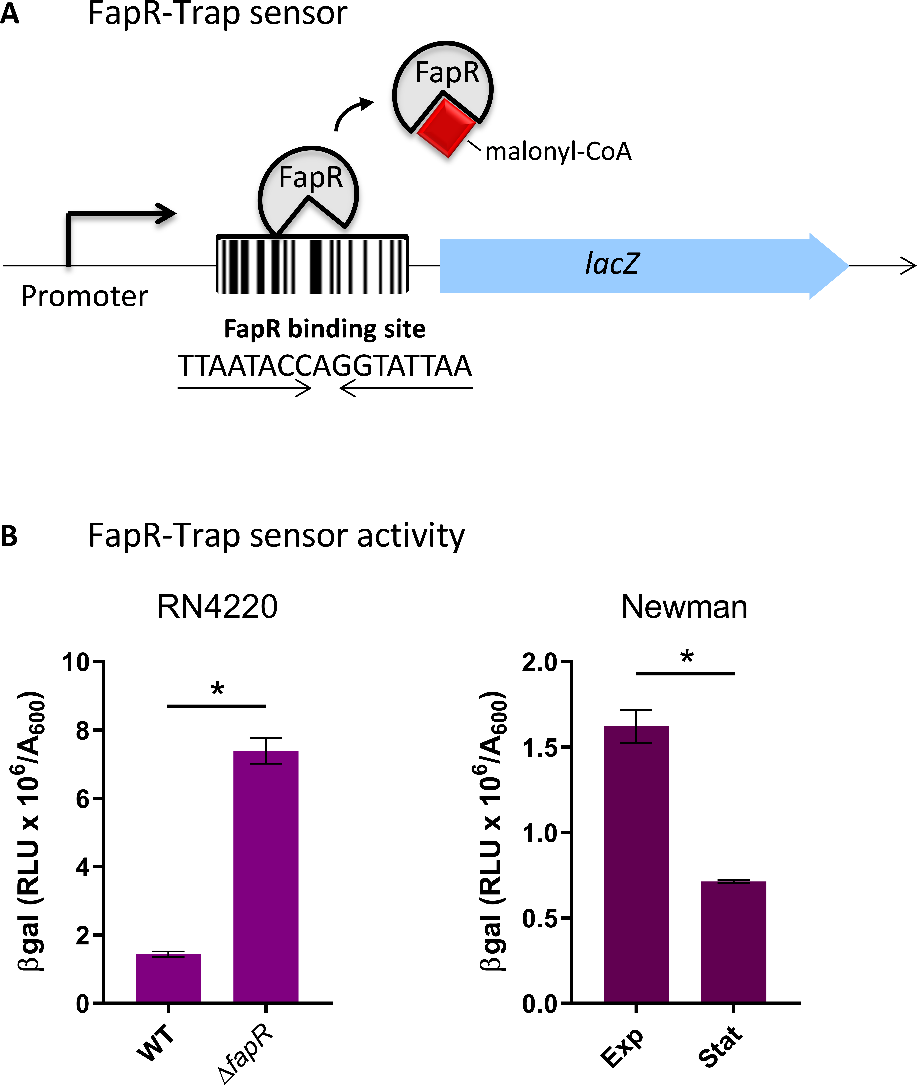


**Supplementary Fig. S3. FapR-Trap, a malonyl-CoA sensor based on FapR operon *lacZ* fusion. A.** Schematic design of FapR-Trap (pJJ004, Table S1). Malonyl-CoA (red diamond) binds FapR (pacman) leading to its release from the FapR binding site (bar code) and expression of *lacZ* (blue) to produce β-galactosidase. The 17 bp FapR consensus binding site used in the construction is shown (based on (1)); converging arrows indicate the 8 bp inverted repeat. **B.** Validation of the FapR-Trap as sensor. β-gal assays were performed with RN4220 and its Δ*fapR* derivative RN4220_Δ_*_fapR_* (1) carrying FapR-Trap after 3 h growth in SerFA (left). FapR-Trap expression was also compared in exponential (Exp) and stationary phase (Stat) of the Newman strain (right). Data presented are mean ± standard deviation from triplicate independent experiments. *, p ≤ 0.05 using Mann Whitney.

1. Albanesi D, Reh G, Guerin ME, Schaeffer F, Debarbouille M, Buschiazzo A, Schujman GE, de Mendoza D, Alzari PM. 2013. Structural basis for feed-forward transcriptional regulation of membrane lipid homeostasis in *Staphylococcus aureus*. PLoS Pathog 9:e1003108.
