# Supplementary material for: (p)ppGpp/GTP and Malonyl-CoA Modulate Staphylococcus aureus Adaptation to FASII Antibiotics and Provide a Basis for Synergistic Bi-Therapy
Source: mBio. 2021 Feb 2;12(1):e03193-20. doi: 10.1128/mBio.03193-20 (PMC7858065; doi:10.1128/mBio.03193-20)
Supplement: TABLE S3 [file mBio.03193-20-st003.docx]

**Table S3.** **Total and proportion of FapR-bound malonyl-CoA depends on growth condition.**

|  | Relative proportions *^a^* | | % Malonyl-CoA bound to FapR |
| --- | --- | --- | --- |
|  | Total Malonyl-CoA pools | FapR-Trap |  |
| Non- selective *^b^* | 100 | 8 | 8% |
| Anti-FASII *^c^*  Latency | 23 | 10 | 43 % |
| Anti-FASII *^c^*  Adapted- Exponential | 93 | 100 | 108 % |

***^a^*** Relative proportions of total and FapR-bound malonyl-CoA from Fig. 3B and 3C are determined based on the highest obtained value for respective measurements (indicated as 100 %). *^b^* Medium was SerFA. *^c^* FASII inhibitor triclosan is added to SerFA (SerFA-Tric).
