# Supplementary material for: (p)ppGpp/GTP and Malonyl-CoA Modulate Staphylococcus aureus Adaptation to FASII Antibiotics and Provide a Basis for Synergistic Bi-Therapy
Source: mBio. 2021 Feb 2;12(1):e03193-20. doi: 10.1128/mBio.03193-20 (PMC7858065; doi:10.1128/mBio.03193-20)
Supplement: TABLE S4 [file mBio.03193-20-st004.docx]

**Table S4. Responses of FapR regulon genes and known stringent response induced genes to mupirocin.**

|  | Promoter fusion | Mupirocin/ No addition *^a^* | Effect |
| --- | --- | --- | --- |
| Control | Pctl *^b^* | 1.1 ± 0.03 | None |
| Stringent response | P*_ilvD_-lacZ* | 2.1 ± 0.42 | Stimulation |
|  | P*_oppB_-lacZ* | 1.87 ± 0.69 | Stimulation |
|  | P*_cshA_-lacZ* | 0.33 ± 0.06 | Repression |
| FapR regulon | P*_accBC_-lacZ* | 0.25 ± 0.04 | Repression |
|  | P*_fapR plsX_-lacZ* | 0.25 ± 0.03 | Repression |
|  | P*_plsC_-lacZ* | 0.34 ± 0.06 | Repression |

*^a^* Measurements (standard deviation) were determined on three independent samples in BHI medium containing or not mupirocin 0.1 µg/ml (described in Materials and Methods). Experiments were performed independently from those presented in Fig. 1. *^b^* Pctl corresponds to the plasmid vector pTCV-lac ([1](#_ENREF_1)), lacking a *lacZ* promoter.

1. Poyart C, Trieu-Cuot P. 1997. A broad-host-range mobilizable shuttle vector for the construction of transcriptional fusions to beta-galactosidase in gram-positive bacteria. FEMS Microbiol Lett 156:193-8.
