# Supplementary material for: (p)ppGpp/GTP and Malonyl-CoA Modulate Staphylococcus aureus Adaptation to FASII Antibiotics and Provide a Basis for Synergistic Bi-Therapy
Source: mBio. 2021 Feb 2;12(1):e03193-20. doi: 10.1128/mBio.03193-20 (PMC7858065; doi:10.1128/mBio.03193-20)
Supplement: TABLE S5 [file mBio.03193-20-st005.docx]

**Table. S5. Subinhibitory mupirocin treatment synergizes with AFN-1252 to inhibits MRSA USA300 growth.** *^a^*

| **Media** | ***A*_600_ at 16 h** |
| --- | --- |
| SerFA | 11.4 ±0.3 |
| SerFA+AFN-1252 ***^b^*** | 11.1 ±0.7 |
| SerFA + Mupirocin | 6.3 ±1.7 |
| SerFA-AFN-1252+Mupirocin | 0.2 ±0.06 |

*^a^* Mupirocin was used at 0.06 µg/ml, AFN-1252 at 0.5 µg/ml. The USA300_FRPR3757 strain is MRSA (methicillin resistant *S. aureus*). It was precultured in SerFA medium, and diluted 1:100 in SerFA containing or not mupirocin and AFN-1252. A_600_ optical densities were determined after 16 h aerobic growth at 37°C. Results shown are the average (range) of 2 independent experiments. *^b^* AFN-1252 is a pipeline antibiotic that, like triclosan, targets FabI ([1](#_ENREF_1)).

1. Banevicius MA, Kaplan N, Hafkin B, Nicolau DP. 2013. Pharmacokinetics, pharmacodynamics and efficacy of novel FabI inhibitor AFN-1252 against MSSA and MRSA in the murine thigh infection model. J Chemother 25:26-31.
