# Supplementary material for: (p)ppGpp/GTP and Malonyl-CoA Modulate Staphylococcus aureus Adaptation to FASII Antibiotics and Provide a Basis for Synergistic Bi-Therapy
Source: mBio. 2021 Feb 2;12(1):e03193-20. doi: 10.1128/mBio.03193-20 (PMC7858065; doi:10.1128/mBio.03193-20)
Supplement: TABLE S6 [file mBio.03193-20-st006.docx]

**Table S6.** **Primers.** *^a^*

| **Primer ID** | **Sequence of Primer (5'to 3')** |
| --- | --- |
| FapRtrapfd | AATTCTCCTACGACAATATCCT**TTGCAT**TTAAAGTATCTAAAATC**TATGAT**AAA***TTAATACCTGGTATTAA***AAATATTTATTAGAAG  *pJJ004 (FapR-Trap)* |
| FapRtraprp | GATCCTTCTAATAAATATTT***TTAATACCAGGTATTAA***TTT**ATCATA**GATTTTAGATACTTTAA**ATGCAA**AGGATATTGTCGTAGGAG  *pJJ004 (FapR-Trap)* |
| ilvDfp | TTGCCGGAATTCCCTATATTATGCTTTTCATTCA  *pJJ005 (P_ilvD_-lacZ)* |
| ilvDrp | CTAGCGGGATCCTTACATGTCGCTTCGCATAGT  *pJJ005 (P_ilvD_-lacZ)* |
| OppBfp | TTGCCGGAATTCTTGAAAAATGGATCATCAGA  *pJJ006 (P_oppB_-lacZ)* |
| OppBrp | CAAGCGGGATCCTTAAATATATTTCCCCATCTAA  *pJJ006(P_oppB_-lacZ)* |
| CshAfp | TTGCCGGAATTCCTTTTACTTATAAAAATGATTTG  *pJJ008 (P_cshA_-lacZ)* |
| CshArp | CAAGCGGGATCCTTATTTAAAATTTTGCAAAATAATTC  *pJJ008 (P_cshA_-lacZ)* |
| FapRfp | TTGCCGGAATTCCAGCTGAACTTATTCAATCTGG  *pJJ013 (P_fapR plsX_-lacZ)* |
| FapRrp | CAAGCGGGATCCTTACGTCTCACCCCTCATTTTTTAGT  *pJJ013 (P_fapR plsX_-lacZ)* |
| PlsCfp | TTGCCCGAATTCAAGTGCACCAATAATTCCAGCA  *pJJ019 (P_plsC_-lacZ)* |
| PlsCrp | CAAGCGGGATCCTTAAATCACTGAATACATTGTGCCACC  *pJJ019 (P_plsC_-lacZ)* |
| AccBCfp | AATTCGTCGCCAGCAATATGAACATGC**TTGAAT**TGAAGAGTTGTCTCAAG**TAAAAT**AGACGGGTAGATGAAAACAAACTGAAGGAGTCAGTAATAATGAACTTTAAAG  *pJJ027 (P_accBC_-lacZ)* |
| AccBCrp | GATCCTTTAAAGTTCATTATTACTGACTCCTTCAGTTTGTTTTCATCTACCCGTCT**ATTTTA**CTTGAGACAACTCTTCA**ATTCAA**GCATGTTCATATTGCTGGCGACG  *pJJ027 (P_accBC_-lacZ)* |
| FapRORFfp | AACTAGCTAGCCATCATCATCATCATCACGAAAACCTGTATTTTCAGGGCATGAGGGGTGAGACGTTGAAACTAAAGAAAG pJJ042 (FapR-ORF) |
| FapRORFrp | AACGCGTCGACTTATCCTCGCTTATCATAAAACATTTTAAAATTTCC  pJJ042 (FapR-ORF) |
| pAW8AccBCfp | TTGCCGGAATTCGTCGCCAGCAATATGAACATGCTTG  pJJ043 (P*accBC-lacZ* for pAW8 insertion) |
| pAW8AccBCrp | TGCGTAAGGAGAAAATACCGCATCAGCCCGGGTTATTTTTGACACCAGACCAACTG  pJJ043 (P*accBC-lacZ* for pAW8 insertion) |
| fakB1_fp | CCTGCAGGTCGACTCTAGAGGATCCGTTCGACACGCCCGATATCA |
| fakB1_rp | ACAGCTATGACATGATTACGAATTCTACCTGCACCTGTTACGGC |
| pG1_GibBam | GGATCCTCTAGAGTCGACCTGCAGG |
| pG1_GibEco | GAATTCGTAATCATGGTCATAGCTG |
| *PfapRplsX*_Fw | CGACTAAATAATAGCTAAATATTACAG |
| P*plsC*_Fw | CAACTTTAGATGTATTTTCAGACTATC |
| pTCV-*lac_*Rev  (vector primer) | CCACAGTAGTTCACCACCTTTTCCC |

*^a^* Restriction sites and overlap sequences in Gibson clonings are underlined; putative -10 and -35 motifs are in bold; FapR consensus binding site is in italics.
