# Supplementary material for: (p)ppGpp/GTP and Malonyl-CoA Modulate Staphylococcus aureus Adaptation to FASII Antibiotics and Provide a Basis for Synergistic Bi-Therapy
Source: mBio. 2021 Feb 2;12(1):e03193-20. doi: 10.1128/mBio.03193-20 (PMC7858065; doi:10.1128/mBio.03193-20)
Supplement: FIG S2 [file mBio.03193-20-sf002.docx]

**Supplementary Fig. S2. Responses of P*_ilvD_-lacZ*, P*_cshA_-lacZ*, and P*_accBC_-lacZ* to mupirocin depend on the presence of (p)ppGpp.** HG1-R is an HG001 derivative repaired for a *fakB1* defect common to the 8325 lineage (1). ppGpp0 is the HGR-1 strain devoid of the three synthase genes *rsh*, *relP*, and *relQ* (2). The indicated strains were plated (1 ml of *A*_600_ = 0.1) on BHI medium containing 100 µg/ml X-gal and 5 µg/ml erythromycin and allowed to dry. Mupirocin (75, 37.5, 18.3, and 9.1 ng in rows starting from upper left) was deposited in 3 µl drops. **A**. Expression of P*_ilvD_*-*lacZ* is induced by mupirocin (Fig. 1A), seen as a blue ring in HG1-R, which is absent in the ppGpp0 strain. **B**. P*_cshA_-lacZ* is repressed by mupirocin (Fig. 1A), seen as a non-blue growth ring, which is quasi-absent in the ppGpp0 strain. **C.** The P*_accBC_-lacZ* sensor behaves like P*_cshA_-lacZ*, indicating that production of malonyl-CoA by ACC is repressed by the stringent response. Plates were photographed after 24 h at 37°C and 24 h at 4°C. Dark zones indicate growth inhibition by mupirocin. The ppGpp0 strain is more sensitive to mupirocin than the isogenic parent. Experiments were performed three times giving comparable results.

1.	Kenanian G, Morvan C, Weckel A, Pathania A, Anba-Mondoloni J, Halpern D, Gaillard M, Solgadi A, Dupont L, Henry C, Poyart C, Fouet A, Lamberet G, Gloux K, Gruss A. 2019. Permissive Fatty Acid Incorporation Promotes Staphylococcal Adaptation to FASII Antibiotics in Host Environments. Cell Rep 29:3974-3982 e4.

2.	Geiger T, Kastle B, Gratani FL, Goerke C, Wolz C. 2014. Two small (p)ppGpp synthases in *Staphylococcus aureus* mediate tolerance against cell envelope stress conditions. J Bacteriol 196:894-902.
